# Supplementary material for: Structure and function of the N‐terminal domain of the human mitochondrial calcium uniporter
Source: EMBO Rep. 2015 Sep 4;16(10):1318–33. doi: 10.15252/embr.201540436 (PMC4662854; doi:10.15252/embr.201540436)
Supplement: Supplementary file 1 — Appendix [file EMBR-16-1318-s001.pdf]

Manuscript EMBOR-2015-40436V2

Structure and function of the N-terminal domain of the human mitochondrial calcium uniporter

Youngjin Lee‡, Choon Kee Min‡, Tae Gyun Kim, Hong Ki Song, Yunki Lim, Dongwook Kim, Kahee Shin, Moonkyung Kang, Jung Youn Kang, Hyung-Seop Youn, Jung-Gyu Lee, Jun Yop An, Kyoung Ryoung Park, Jia Jia Lim, Ji Hun Kim, Ji Hye Kim, Zee Yong Park, Yeon-Soo Kim, Jimin Wang, Do Han Kim* and Soo Hyun Eom*

Corresponding author: Do Han Kim and Soo Hyun Eom, Gwangju Institute of Science and Technology, GIST

Appendix

Table of Contents

Appendix Materials and Methods 2−5

Appendix Figures

Appendix Figure S1: Multiple sequence alignment of MCUs. 6−7

Appendix Figure S2: An unidentified lipid like molecule of MCU NTD-E. 8

Appendix Figure S3: Cross-linking of MCU NTD-E_{WT} 9

Appendix Figure S4: Circular dichroism analysis of $MCU_{\Delta NTD}$, MCU NTD-E_{WT} and mutants 10−11

Appendix Figure S5: Co-immunopricipitation of MCU_{WT} and $MCU_{\Delta NTD}$ 11−12

Appendix Figure S6: Generation of mitochondrial targeting GGECO and simultaneous cytosolic and mitochondrial Ca^{2+} imaging. 13

Appendix Tables

Appendix Table S1: Dali search result of MCU NTD. 14

Appendix Table S2: CATH search result of MCU NTD. 15

Appendix References 16

Appendix Material and Methods

Cloning of MCUR1 constructs

The sequences encoding MCUR1 (NP_001026883) residues 138–338 ($MCUR1_{138–338}$) were amplified using polymerase chain reaction (PCR) from human oral squamous carcinoma YD-10B cDNA. For *in vitro* binding assays, $MCUR1_{138–338}$ was cloned into a modified pET28a vector (Novagen) containing His_6-maltose binding protein (MBP)-TEV. Each sequence was also cloned into modified pET41a vector (Novagen) containing glutathione S-transferase (GST), which altered the thrombin site for a TEV protease site.

For mammalian cell expression, full-length MCUR1 was cloned into pCS2-MT vector, which also encodes an N-terminal 6 × myc. pCS2-MT vector was purchased from 21C Frontier Human Gene Bank (Daejeon, Korea).

In vitro binding of MCU to MCUR1

His- or GST-fused MCU NTD and MCUR1 (residues, 138–338; $MCUR1_{138–338}$) were purified using conventional Ni-NTA or GST affinity methods followed by SEC using a final buffer containing 20 mM Tris-HCl (pH 7.5), 100 mM NaCl, 0.5 mM EDTA, 0.5 mM EGTA, and 0.5 mM DTT. All reactive proteins were combined to final level of 0.25 mg per GST column pre-packed with 0.3 mL of GST beads. His-MCU NTD and GST-$MCUR1_{138–338}$ or His-MBP-$MCUR1_{138–338}$ and GST-MCU NTD with GST resin were incubated for 12 h on ice. To visualize pull-down combinations, samples were subjected to 20% (*w/v*) SDS-PAGE and western blotting as described below.

The blotting step was preceded by 20% SDS-PAGE (200 V and 100 mA) for at least 2 h and transfer to PVDF membranes using a Bio-Rad transfer system. Membranes were blocked with blocking buffer (5% skim milk powder, 25 mM Tris-HCl, and 192 mM glycine and 0.1% Tween-20) for 2 h at room temperature and incubated overnight at 4°C with primary anti-His and anti-GST antibodies in blocking buffer. Membranes were then washed three times with blocking buffer for 5 min at room temperature and incubated with rabbit polyclonal antibody (Promega) for 2 h at room temperature with gently shaking in blocking buffer. Finally, membranes were again washed three times with washing buffer [25 mM Tris-HCl pH 8.0, and 192 mM glycine, and 0.2% Tween-20] and visualized using BCIP (16.5 μL) and NBT (33 μL) in developing buffer [20 mM Tris-HCl pH 8.0, 100 mM NaCl, and 1 mM $MgCl_2$].

Cross-linking assay

To confirm the oligomerization of MCU NTD-E, cross-linking assay was performed using glutaraldehyde (GA) as a cross-linker. MCU NTD-E was prepared in phosphate-buffered saline (PBS) (pH 7.4) as well as crystallization buffer containing equal volumes of protein [20 mM Tris-HCl (pH 7.8), 100 mM NaCl, 5% glycerol, 1 mM DTT] and the reservoir solution [1.55 M lithium sulphate and 0.1 M Bis-Tris propane (pH 8.0)]. GA was treated to MCU NTD-E proteins at different concentration of 0, 0.003, 0.01, and 0.03%. The samples were reacted at 4°C for 2 h and the reaction was stopped by additional 1 M Tris-HCl (pH 8.5). The samples with 5 x reduced sample buffer were then boiled and were subjected to 17% (*w/v*) SDS-PAGE to visualize the cross-linking results.

Circular dichroism (CD) analysis

1.0 mg mL^{-1} proteins of MCU NTD-E wild type (NTD-E_{WT}) and each mutants, S92A (NTD-E_{S92A}) and K180A (NTD-E_{K180A}), were prepared using the same cloning and purification methods of MCU NTD-E_{WT} for crystallization.

$MCU_{\Delta NTD}$ (residues, 166−351) was cloned into the pET21a vector (Novagen) and expressed in *Escherichia coli* strain BL21 (DE3). Transformed cells were cultured in Luria-Bertani medium containing 100 μg mL^{-1} ampicillin at 37°C. After addition of 0.5 mM IPTG (Goldbio), the cells were incubated at 20°C for an additional 20 h. After sonication, the cells were resuspended in lysis buffer [50 mM Na-phosphate (pH 7.4), 300 mM NaCl, 5 mM imidazole, 1 mM PMSF], the membranous fraction was collected by centrifugation (1 h at 32000g) and solubilized with 2.0% (*w/v*) n-dodecyl-β-D-maltopyranoside (DDM) for 3 h at 4°C. The non-solubilized material was removed by centrifugation and the supernatant was loaded on a nickel chromatography column (Elpis). Column was washed with wash buffer [50 mM Tris-HCl (pH 7.8), 300 mM NaCl, 50 mM imidazole] and the protein was eluted with 500 mM imidazole. Fractions of 30 mL were collected and dialysed against CD analysis buffer [20 mM Tris-HCl (pH 7.8), 50 mM KCl, 1 mM DTT, 1 mM EGTA, 1 mM EDTA, 0.05% (*w/v*) DDM]. A concentration of 0.05% DDM was maintained throughout the purification.

1.0 mg mL^{-1} proteins of MCU NTD-E_{WT}, NTD-E_{S92A}, and NTD-E_{K180A} in final buffer [20 mM Tris-HCl (pH 7.8), 50 mM KCl, 1 mM EDTA, 1 mM EGTA, and 1 mM DTT] and 0.1 mg mL^{-1} proteins of $MCU_{\Delta NTD}$ in CD analysis buffer were used for CD analysis. CD spectra of the proteins were recorded on a Jasco J-815 spectrometer over the wavelength range of 190−260 nm in a 1.0-mm path length quartz cuvette with 1.0 nm bandwidth. Each spectrum represents the accumulation of four scans. The temperature of the cuvette was maintained

at 20°C. Secondary structure of $MCU_{\Delta NTD}$ *w*as examined using Dichroweb software package [1] containing SELCON3 [2], CONTIN, CDSSTR [3], and K2D [4]. Secondary structure of $MCU_{\Delta NTD}$ was predicted by PredictProtein server and Network Protein Sequence Analysis server (NPS@) using GOR1, GOR3, HNNC, MLRC, PHD, SOPM, and Predator parameters [5].

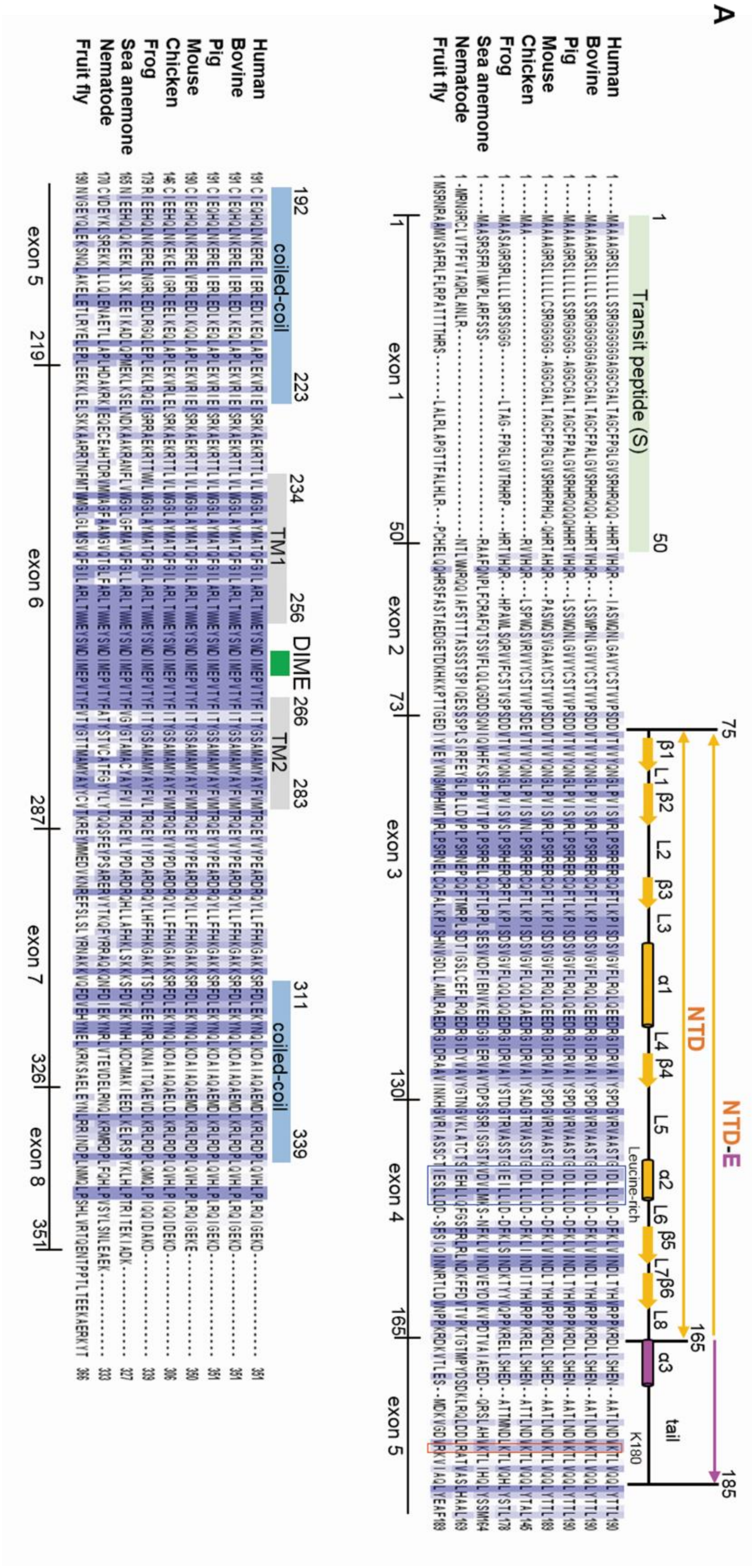
A
Human
Bovine
Pig
Mouse
Chicken
Frog
Sea anemone
Nematode
Fruit fly
Transit peptide (S)
NTD
NTD-E
Leucine-rich
tail
K180
exon 1
exon 2
exon 3
exon 4
exon 5
exon 6
exon 7
exon 8
coiled-coil
TM1
TM2
DIME

B

Species	NCBI accession number	Identity (%) (Full / NTD)	Homology (%) (Full / NTD)
Human (*Homo sapiens*)	NP_612366.1	-	-
Bovine (*Bos taurus*)	NP_001193031.1	98.6/ 100.0	99.2/ 100.0
Pig (*Sus scrofa*)	XM_001925826.4	98.3/ 100.0	98.9/ 100.0
Mouse (*Mus musculus*)	NP_001028431.2	96.0/ 100.0	98.0/ 100.0
Chicken (*Gallus gallus*)	XP_004942087.1	77.5/ 91.2	83.2/ 95.6
Frog (*Xenopus tropicalis*)	NP_001123699.1	75.7/ 84.6	86.4/ 94.5
Sea anemone (*Nematostella vectensis*)	XP_001624088.1	47.2/ 51.7	67.2/ 76.9
Nematode (*Caenorhabditis elegans*)	NP_500892.1	35.5/ 45.1	58.9/ 72.5
Fruit fly (*Drosophila melanogaster*)	NP_996003.1	39.7/ 55.0	62.2/ 69.2

Appendix Figure S1. Multiple sequence alignment of MCUs.

(A) The amino acid sequence alignment of MCUs from various species is listed in **B**. β-strands (β1−β6) and α-helices (α1−α3) are represented by arrows and cylinders, respectively. K180, the ubiquitination site, is highlighted by a red box and leucine-rich (LR) regions ($_{141}$IDLLLL$_{146}$) are indicated by a blue box. The MCU NTD and NTD-E regions are marked by arrows. Other regions, including the transmembrane domains (TM1 and TM2), "DIME" motif, two coiled-coils, and transit peptide (S) are represented by grey, dark green, dark blue, and light green rectangles, respectively. Exons (1−8) of human MCU are represented by black lines.

(B) Sequence identity and homology analysis with various MCUs indicates that MCU NTD is highly conserved.

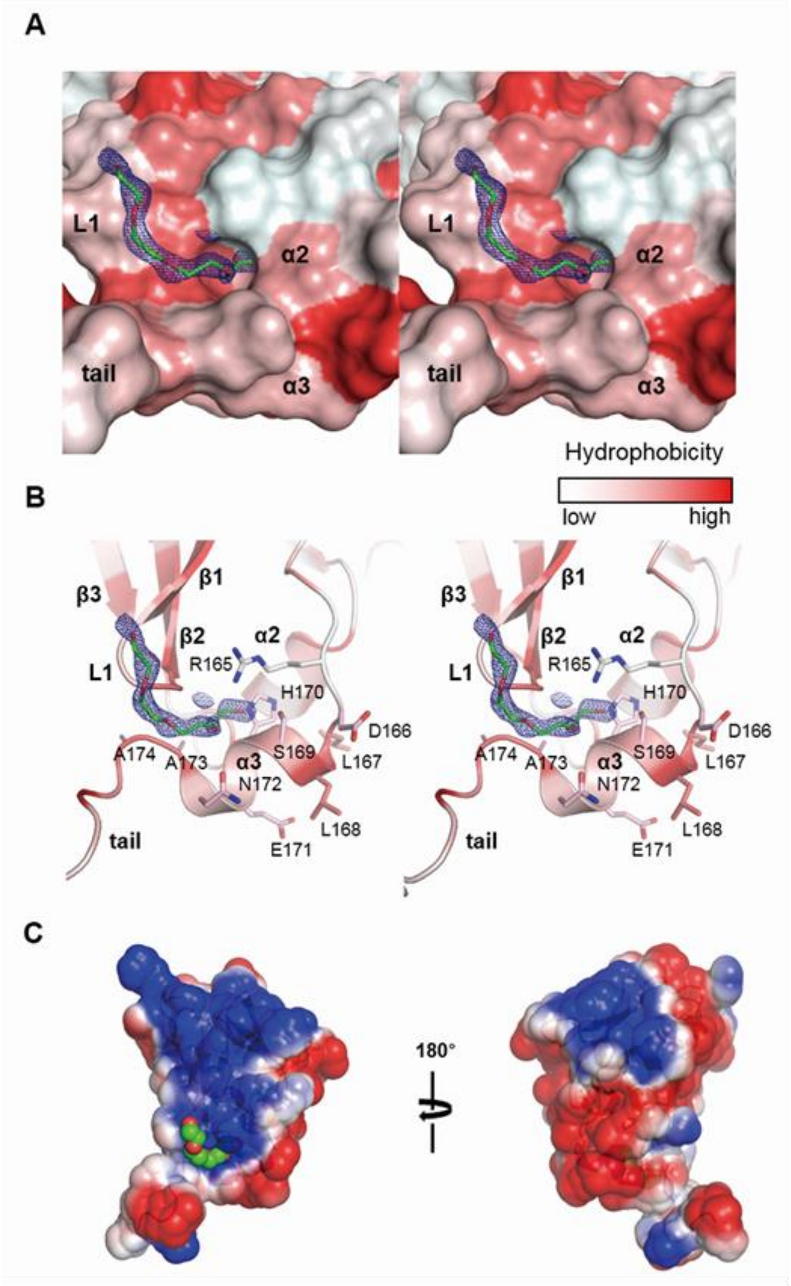

Appendix Figure S2. An unidentified lipid like molecule of MCU NTD-E.

(A) Stereoview of MCU NTD-E hydrophobic surface with the unidentified lipid-like molecule fitted to a tetraethylene glycol molecule. The sigma-weighted 2*F*o-*F*c electron density map contoured at 1.2 σ is described as blue mesh.

(B) Stereo-cartoon view of MCU NTD-E including the unidentified lipid-like molecule. The molecule located to hydrophobic pocket among L1 loop, two helices (α2 and α3), and tail.

(C) Surface charge of MCU NTD-E.

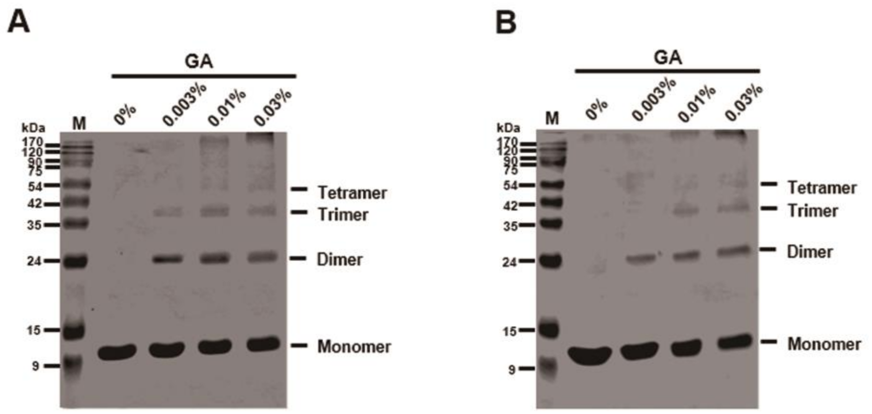

Appendix Figure S3. Cross-linking of MCU NTD-E.

(A, B) *In vitro* cross-linking of MCU NTD-E. The MCU NTD-E proteins were reacted for 2 h at 4°C with various glutaraldehyde (GA) concentration (0, 0.003, 0.01, and 0.03%) in PBS (**A**) as well as crystallization buffer (**B**). The results were generated by 17% (*w/v*) SDS-PAGE analysis (Coomassie blue stained).

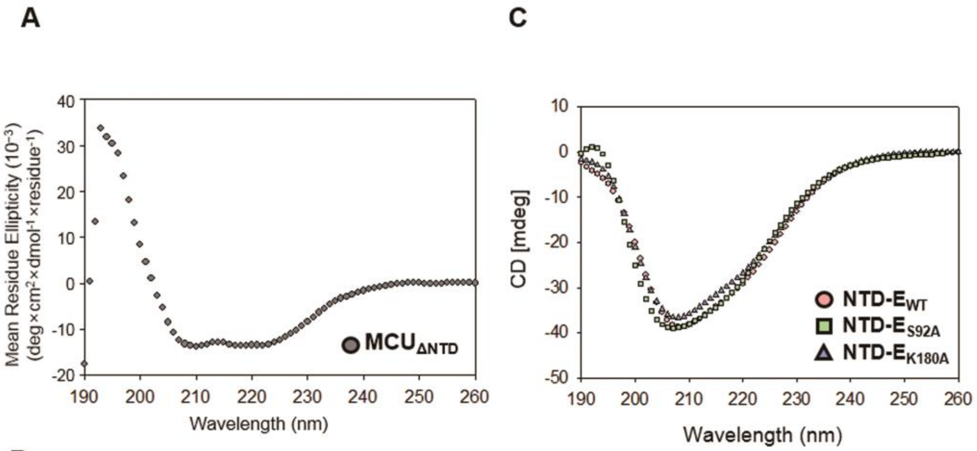

B

Sample	Method	α-helix (%)	β-sheet (%)	coil (%)
$MCU_{\Delta NTD}$	CD analysis*	60.8 ± 10.1	12.2 ± 3.9	28.8 ± 12.8
	Secondary structure prediction†	66.4 ± 12.6	11.5 ± 11.5	23.6 ± 15.9

* Secondary structure of $MCU_{\Delta NTD}$ *was* examined using Dichroweb software package [1] containing SELCON3 [2], CONTIN, CDSSTR [3], and K2D [4].
† Secondary structure of $MCU_{\Delta NTD}$ was predicted by PredictProtein server and Network Protein Sequence Analysis server (NPS@) using GOR1, GOR3, HNNC, MLRC, PHD, SOPM, and Predator parameters [5].

Appendix Figure S4. Circular dichroism analysis of $MCU_{\Delta NTD}$, MCU NTD-EWT and mutants.

(A) Circular dichroism (CD) measurements for $MCU_{\Delta NTD}$ (grey circle) at 190–260 nm ultraviolet (UV) wavelength. The x-axis and y-axis of the graph describe the UV wavelength and mean residue ellipticity. The spectrum indicates the average of 4 replicate scans.

(B) Secondary structure prediction of $MCU_{\Delta NTD}$. The spectrum of $MCU_{\Delta NTD}$ represents canonical α-helical profiles with minima at 208 and 222 nm. Secondary structure of $MCU_{\Delta NTD}$ was examined using Dichroweb software package and predicted by PredictProtein server and Network Protein Sequence Analysis server (NPS@) (see the footnote). The helical contents of predicted CD spectrum and secondary structure prediction based on $MCU_{\Delta NTD}$ protein sequence are 61 and 66%, respectively.

(C) CD measurements for MCU NTD-E_{WT} (red circle) and mutants, NTD-E_{S92A} (green

rectangular) and NTD-E_{K180A} (blue triangular) at 190–260 nm ultraviolet (UV) wavelength. The x-axis and y-axis of the graph describe the UV wavelength and raw CD spectrum data. Each spectrum indicates the average of 4 replicate scans.

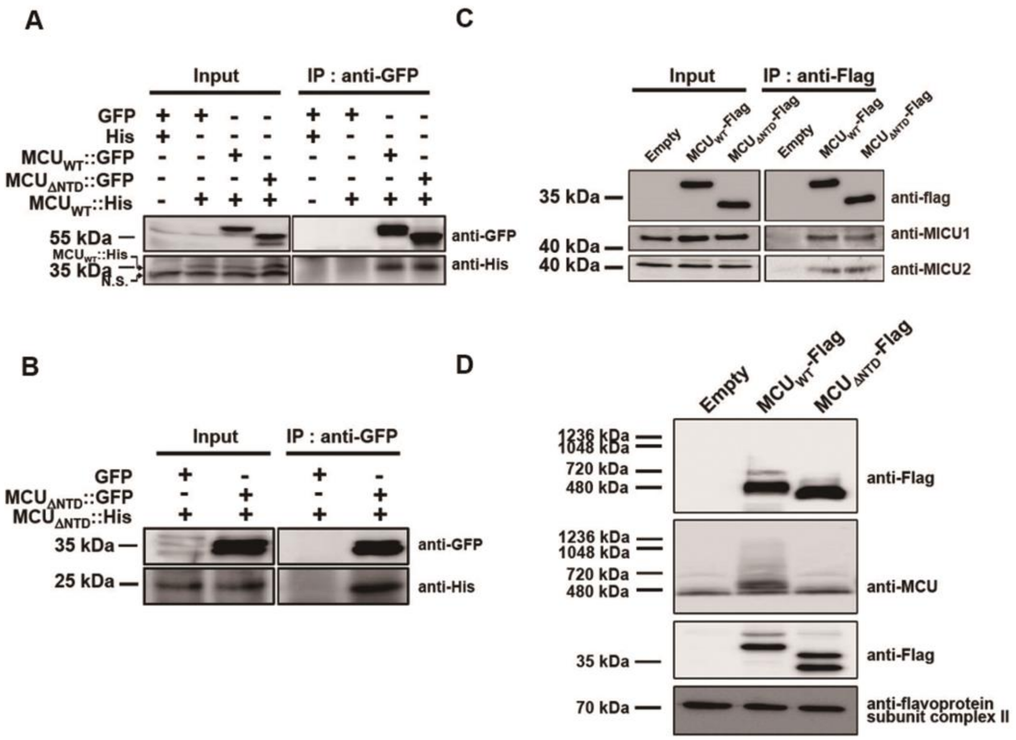

Appendix Figure S5: Co-immunopricipitation of MCU_{WT} and $MCU_{\Delta NTD}$.

(A) Co-immunoprecipitation of MCU_{WT}/$MCU_{\Delta NTD}$-GFP with His-MCU_{WT}. HEK-293 FT cells were transiently co-transfected with His-MCU_{WT} and MCU_{WT}/$MCU_{\Delta NTD}$-GFP. $MCU_{\Delta NTD}$ migrated farther than MCU_{WT}, indicating an apparent molecular weight difference of 9 kDa in SDS-PAGE. MCU_{WT}-GFP and $MCU_{\Delta NTD}$-GFP were precipitated from the cell lysates with an anti-GFP antibody. The precipitates were separated on SDS-PAGE and immunoblotted with the antibodies indicated.

(B) Co-immunoprecipitation of $MCU_{\Delta NTD}$-GFP with His-$MCU_{\Delta NTD}$. HEK-293 FT cells were transiently co-transfected with His-$MCU_{\Delta NTD}$ and $MCU_{\Delta NTD}$-GFP. $MCU_{\Delta NTD}$-GFP was precipitated from cell lysates with an anti-GFP antibody. The precipitates were separated on SDS-PAGE and immunoblotted with the indicated antibodies.

(C) Interaction of $MCU_{\Delta NTD}$ with MICU1 and MICU2 is not altered. After expression of flag-tagged MCU_{WT} and $MCU_{\Delta NTD}$ in HEK293 FT cells, co-immunoprecipitation was performed. The precipitates were subjected to SDS-PAGE and immunoblotting with the indicated antibodies.

(D) HEK-293 FT cells were transiently transfected with MCU_{WT}-Flag or $MCU_{\Delta NTD}$-Flag. After isolation and solubilization of crude mitochondria, the lysates were subjected to BN-PAGE and immunoblotted with anti-Flag and commercially available anti-MCU antibodies (Sigma) to detect ectopic MCU_{WT}-Flag, $MCU_{\Delta NTD}$-Flag, and endogenous MCU. MCU_{WT} and $MCU_{\Delta NTD}$ were detected at apparent molecular weights of 480 and 440 kDa, respectively. The shift shown in $MCU_{\Delta NTD}$ complex correlated with the difference in molecular weight, if it is assumed to be a tetramer.

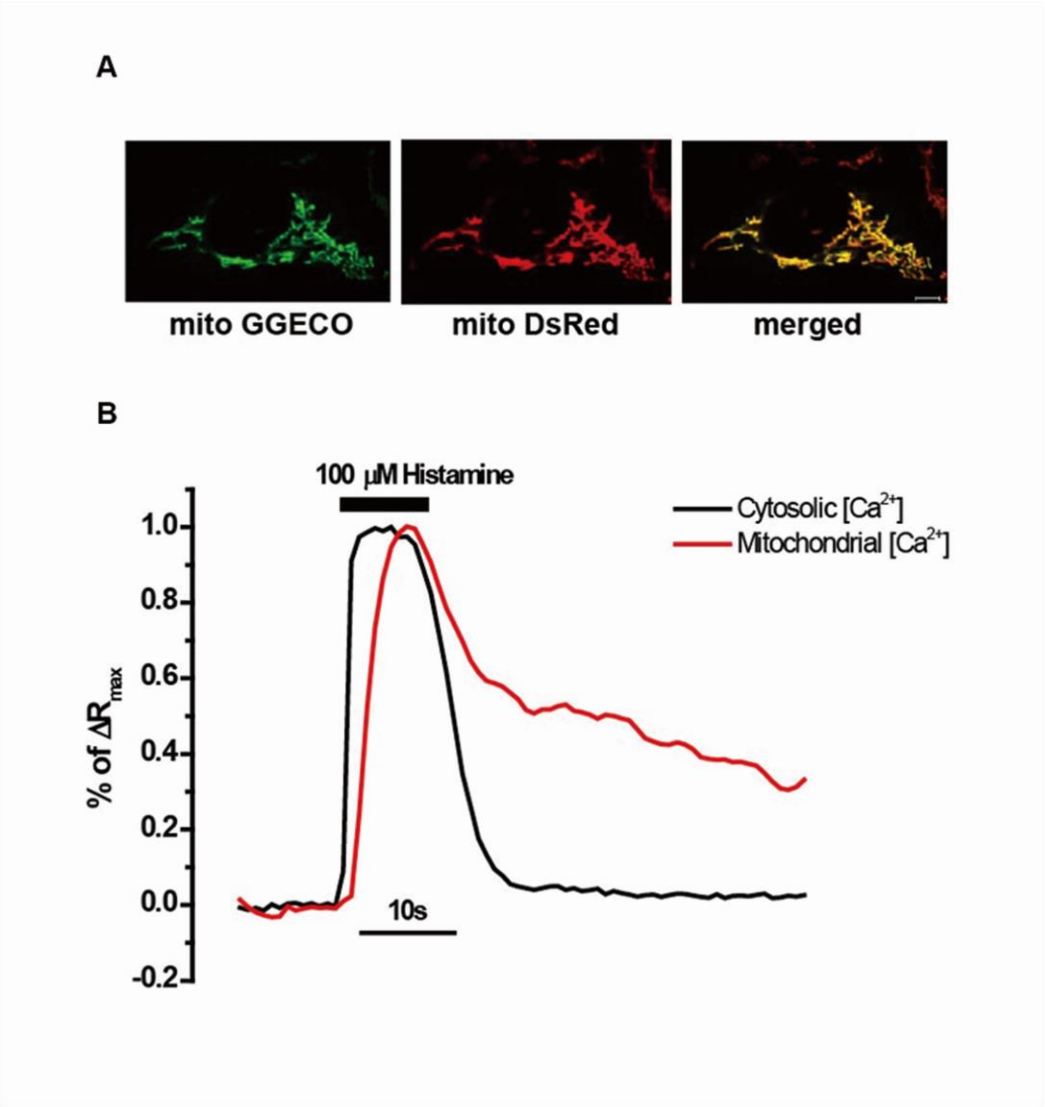

Appendix Figure S6. Generation of mitochondrial targeting GGECO and simultaneous cytosolic and mitochondrial Ca^{2+} imaging.

(A) Localization of mitochondrial targeting GGECO (mito-GGECO) in the mitochondria was confirmed by visualization using a confocal microscope after co-transfection of HeLa cells with mito-GGECO and mito-DsRed (scale bar: 10 μm).

(B) Representative traces of cytosolic and mitochondrial [Ca^{2+}] transients evoked by treatment with 100 μM histamine. The traces were measured by recording the fluorescence intensities of RGECO and mito-GGECO.

Appendix Table S1: Dali search result of MCU NTD.

No*	PDB-Chain	z-score	RMSD (Å)	PDB description
1	3rt3-B	5.8	3.2	Ubiquitin-like protein ISG15
2	3phx-B	5.8	3.2	RNA-directed RNA polymerase L
3	4pyu-B	5.8	2.9	Ubiquitin-like protein 5
7	3b0a-A	5.8	3.5	Polyubiquitin-C
9	1z2m-A	5.8	3.3	Interferon, α-inducible protein
12	3sdl-D	5.7	3.3	Non-structural protein 1
14	3plu-B	5.7	3.1	Ubiquitin-like modifier HUB1
18	4naw-A	5.6	3	Ubiquitin-like protein ATG12
23	3pse-B	5.6	3.3	RNA Polymerase
25	3m62-B	5.6	3.1	Ubiquitin conjugation factor E4
26	4hpl-B	5.5	3.1	Bcl-6 corepressor
27	3w1s-C	5.5	2.7	Autophagy protein 5
28	3dvn-X	5.5	3.2	Human IGG1 Fab fragment light chain
29	2zcb-B	5.5	3.2	Ubiquitin
31	1p3q-V	5.5	3.1	Vacuolar protein sorting-associated protein VPS9
36	3w1y-B	5.4	3.1	Microtubule-associated protein 1A/1B, light chain
41	3r66-C	5.4	3.3	Non-structural Protein 1
43	2zvo-A	5.4	3	UBC protein
47	3dcg-A	5.4	3.2	Transcription elongation factor B polypeptide 2
48	1vcb-A	5.4	3.3	Protein (elongin B)
52	2fuh-B	5.4	2.9	Ubiquitin-conjugating enzyme E2 D3
53	3dvn-U	5.4	3.2	Human IGG1 Fab fragment light chain
55	2bkr-B	5.4	3.1	Sentrin-specific protease 8
57	1f9j-B	5.4	3.2	Tetra-ubiquitin
60	1wr6-F	5.4	3	ADP-ribosylation factor binding protein GGA3
61	2zeq-A	5.4	3.5	E3 ubiquitin-protein ligase parkin
65	4ksl-L	5.4	3.4	Protein FAM105B
66	4rf0-B	5.4	3.1	ORF1AB Protein
87	4n9f-X	5.3	3.2	Cullin-5
89	3bin-A	5.3	3.2	Band 4.1-like protein 3

*Top 30 solutions above Z-score 5.3 are listed.

Appendix Table S2: CATH search result of MCU NTD.

Domain*	PDB-chain	Residues	Superfamily	Description	RMSD (Å)	SSAP score (1–100)
3ge3C00	3GE3-C	83	3.10.20.270	Ubiquitin-like (UB roll) _TmoB-like	7.2	79.3
1wx8A00	1WX8-A	96	3.10.20.90	Ubiquitin-like (UB roll) _phosphatidylinositol 3-kinase catalytic subunit	9.7	76.2
1uh6A00	1UH6-A	100	3.10.20.90	Ubiquitin-like (UB roll) _phosphatidylinositol 3-kinase catalytic subunit	6.9	75.8
3cwiA00	3CWI-A	67	3.10.20.30	Ubiquitin-like (UB roll) _thiamin biosynthesis sulfur carrier protein	5.4	74.1
1mg4A00	1MG4-A	101	3.10.20.230	Ubiquitin-like (UB roll) _doublecortin (DC)	5.4	73.7
1h3fA03	1H3F-A	79	3.10.290.10	Structural genomics hypothetical 15.5 kDa protein in mrca-pcka intergenic region	5.0	73.0
2dnfA01	2DNF-A	89	3.10.20.230	Ubiquitin-like (UB roll) _doublecortin (DC)	9.1	72.9
1xakA00	1XAK-A	68	2.60.40.1550	Immunoglobulin-like _sars orf7a accessory protein	5.7	72.8
1v5vA03	1V5V-A	75	2.40.30.110	Elongation Factor Tu_ aminomethyltransferase β-barrel domains	8.7	72.1
1lxmA04	1LXM-A	76	2.60.40.1380	Immunoglobulin-like _E set domains	5.2	71.9
1y8qD03	1Y8Q-D	105	3.10.290.20	Ubiquitin-like 2 activating enzyme e1b	3.5	71.9
1o75A04	1O75-A	82	2.60.40.1270	Immunoglobulin-like	4.8	71.7
2qdzA03	2QDZ-A	73	3.10.20.310	Ubiquitin-like (UB roll) _membrane protein fhac	7.5	71.7
2v33A00	2V33-A	91	2.60.40.350	Immunoglobulin-like _structural polyprotein	4.1	71.6
1igdA00	1IGD-A	61	3.10.20.10	Ubiquitin-like (UB roll) _immunoglobulin G-binding protein G	9.3	71.3
1im3D00	1LM3-D	95	2.60.40.1200	Immunoglobulin-like _membrane glycoprotein US2	4.9	71.2
2odxA00	2ODX-X	54	2.60.260.40	HSP40/DNAj peptide-binding domain _q5lls5 like domains	6.1	71.1
4e74A00	4E74-A	100	3.10.20.90	Ubiquitin-like (UB roll) _phosphatidylinositol 3-kinase catalytic subunit	6.8	71.0
1op4A01	1OP4-A	89	2.60.40.60	Immunoglobulin-like _cadherins	7.6	70.9
1ok8A04	1OK8-A	100	2.60.40.350	Immunoglobulin-like _genome polyprotein	6.0	70.8
1p9kA00	1P9K-A	79	3.10.290.10	Structural genomics hypothetical 15.5 kDa protein in mrca-pcka intergenic region	8.3	70.5

* Top 21 solutions above SSAP score 70.5 are listed.

Appendix References

1. Whitmore L, Wallace BA. (2008) Protein secondary structure analyses from circular dichroism spectroscopy: methods and reference databases. *Biopolymers* **89**: 392−400

2. Sreerama N, Venyaminov SY, Woody RW (2000) Estimation of protein secondary structure from circular dichroism spectra: inclusion of denatured proteins with native proteins in the analysis. *Anal Biochem* **287**: 243−251

3. Sreerama N, Woody RW (2000) Estimation of protein secondary structure from circular dichroism spectra: comparison of CONTIN, SELCON, and CDSSTR methods with an expanded reference set. *Anal Biochem* **287**: 252−260

4. Andrade MA, Chacón P, Merelo JJ, Morán F (1993) Evaluation of secondary structure of proteins from UV circular dichroism spectra using an unsupervised learning neural network. *Protein Eng* **6**: 383−390

5. Combet C, Blanchet C, Geourjon C, Deléage G (2000) NPS@: network protein sequence analysis. *Trends Biochem Sci* **25**: 147−150
