# Supplementary material for: Structure and function of the N‐terminal domain of the human mitochondrial calcium uniporter
Source: EMBO Rep. 2015 Sep 4;16(10):1318–33. doi: 10.15252/embr.201540436 (PMC4662854; doi:10.15252/embr.201540436)
Supplement: Supplementary file 2 — Expanded View Figures PDF [file EMBR-16-1318-s002.pdf]

Expanded View Figures

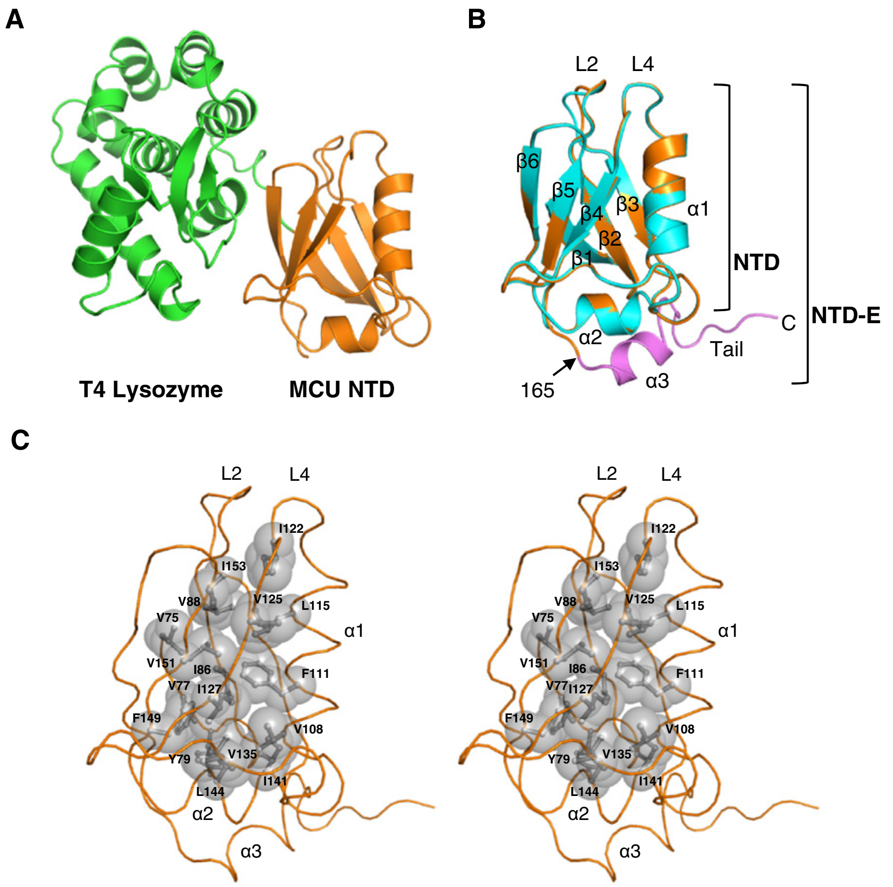

Figure EV1. Detailed structure of MCU NTD.

A Structure of MCU NTD fused with T4 lysozyme at its N-terminus. MCU NTD is shown in orange, while bacteriophage T4 lysozyme is represented in green.

B Superposition of the MCU NTD and MCU NTD-E structures. Superposition of the two structures revealed a root mean square deviation (RMSD) of 0.29 Å for 90 Cα atoms. Two MCU NTDs are shown in cyan for T4 lysozyme-MCU NTD and in orange for MCU NTD-E. Extended C-terminal residues in MCU NTD-E comprising residues 166–182 are shown in magenta.

C Stereoview of the hydrophobic interior of MCU NTD-E. The hydrophobic residues are shown as a grey surface.

© 2015 The Authors

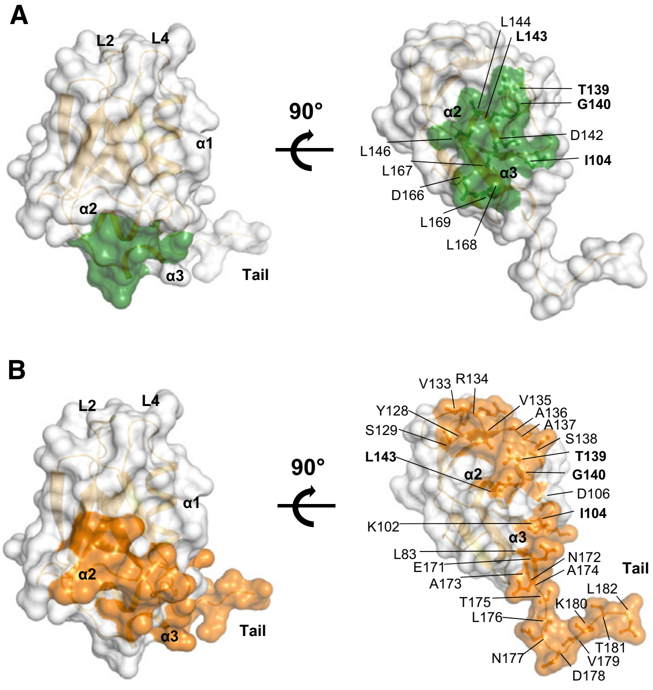

C

Server •		Residues†
InterProSurf	L3 loop	**I104**,
	L5 loop	**T139**
	α2 helix	**G140**, D142, **L143**, L144, L146
	α3 helix	D166, L167, L168, S169
cons-PPISP	β2 sheet	L83
	L3 loop	K102, **I104**, D106
	β4 sheet	Y128
	L5 loop	S129, V133, R134, V135, A136, A137, S138, **T139**,
	α2 helix	**G140**, **L143**,
	α3 helix	E171, N172, A173,
	C-term tail	T175, L176, N177, D178, V179, K180, T181, L182,

• Severs to predict protein-protein interaction (PPI) site.
† Residues of MCU NTD-E predicted by InterProSurf [32] and cons-PPISP [33].

Figure EV2. Prediction of the surface involved in protein–protein interaction (PPI).

A The potential PPI interface is shown as a green surface, as predicted by the InterProSurf server [32]. The residues listed by the InterProSurf analysis show ~70% accuracy for the prediction of the PPI interface, based on the accessible surface area (ASA). Each cluster of selected surface residues was ranked according to its scoring function.

B The potential PPI interface is shown as an orange surface, as predicted by the consensus Protein–Protein Interaction Site Predictor (cons-PPISP) server [33]. The residues listed by cons-PPISP analysis show 80% accuracy with 51% coverage through sequence profiles and solvent accessibility.

C List of residues shown in (A, B).

 © 2015 The Authors

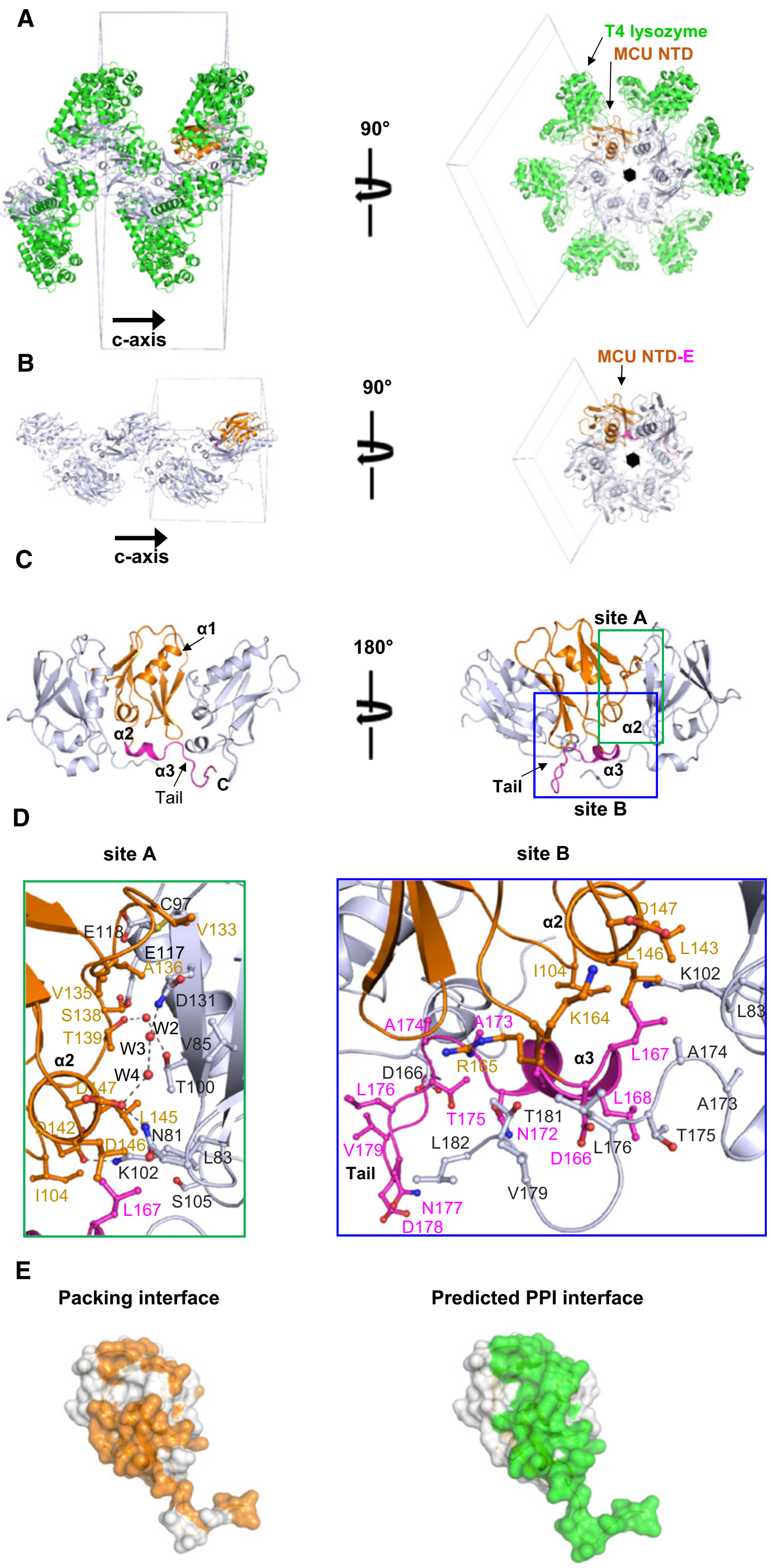

Figure EV3. T4 lysozyme-MCU NTD and MCU NTD-E crystallographic packing.

A, B Oligomers in crystal lattice of T4 lysozyme-MCU NTD (A) and MCU NTD-E (B). MCU NTDs form helical oligomers around the 6_5 screw axis in a similar manner. Continuous domain swapping by C-terminal tail forms oligomer along c-axis in the crystal lattice of MCU NTD-E (B).

C Ribbon diagram of MCU NTD-E oligomer in the crystal. A protomer (in orange) interacts with adjacent molecules through MCU NTD and C-terminus tail.

D Zoomed-in view of the interfaces.

E Crystal packing and PPI interfaces. Interface involved in the crystal packing matches with predicted PPI surface (InterProSurf and cons-PPISP) (Fig EV2).

© 2015 The Authors

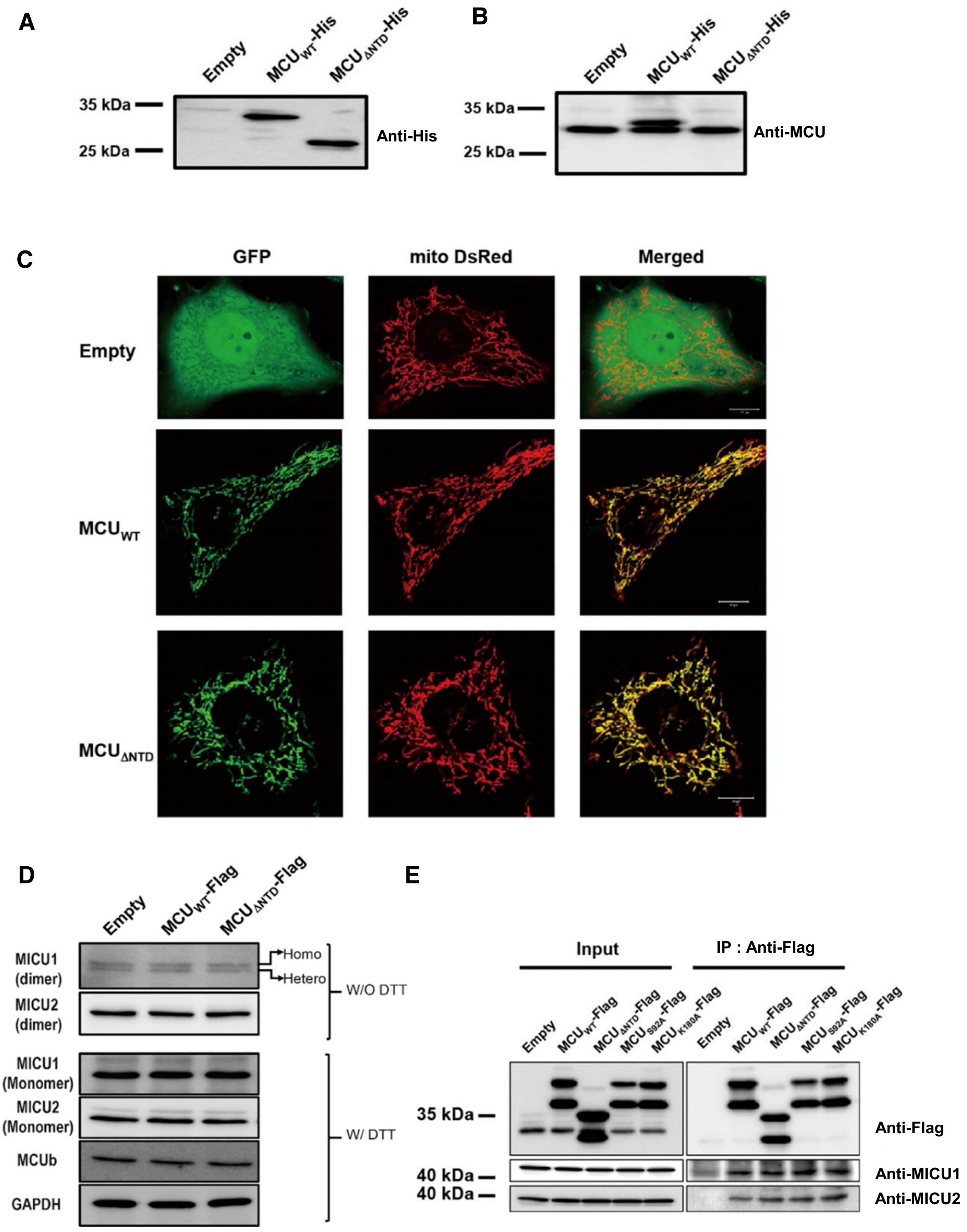

Figure EV4. Expression of MCU$_{WT}$ and MCU mutants in HeLa cells.

A, B His-tagged MCU$_{WT}$ and MCU$_{\Delta NTD}$ were expressed in HeLa cells, and the expression of the proteins was detected with anti-His (A) and anti-MCU (B) antibodies, respectively.

C Localization of MCU$_{WT}$ and MCU$_{\Delta NTD}$ was examined by confocal imaging after co-transfection of GFP-tagged MCU$_{WT}$ or MCU$_{\Delta NTD}$ with mito-DsRed. The merged images indicated that MCU$_{\Delta NTD}$ is localized in the mitochondria, similar to MCU$_{WT}$. Scale bars, 10 μm.

D Expression profiles of MCU regulatory proteins. Twenty micrograms of the lysates with or without dithiothreitol (DTT) were subjected to SDS–PAGE and immunoblotted using the indicated antibodies. The results revealed that the expression levels of the indicated proteins did not change in MCU$_{\Delta NTD}$-overexpressing HeLa cells.

E Interaction of MCU mutants with MICU1 and 2. MCU$_{\Delta NTD}$, MCU$_{S92A}$ and MCU$_{K180A}$ with MICU1 and MICU2 bind to MICU1 and MICU2 as MCU$_{WT}$ does. After expression of Flag-tagged MCU$_{WT}$, MCU$_{\Delta NTD}$, MCU$_{S92A}$ and MCU$_{K180A}$ in MCU-KD HeLa cells, co-immunoprecipitation assay was performed. The precipitates were subjected to SDS–PAGE and immunoblotted with the indicated antibodies.

 © 2015 The Authors

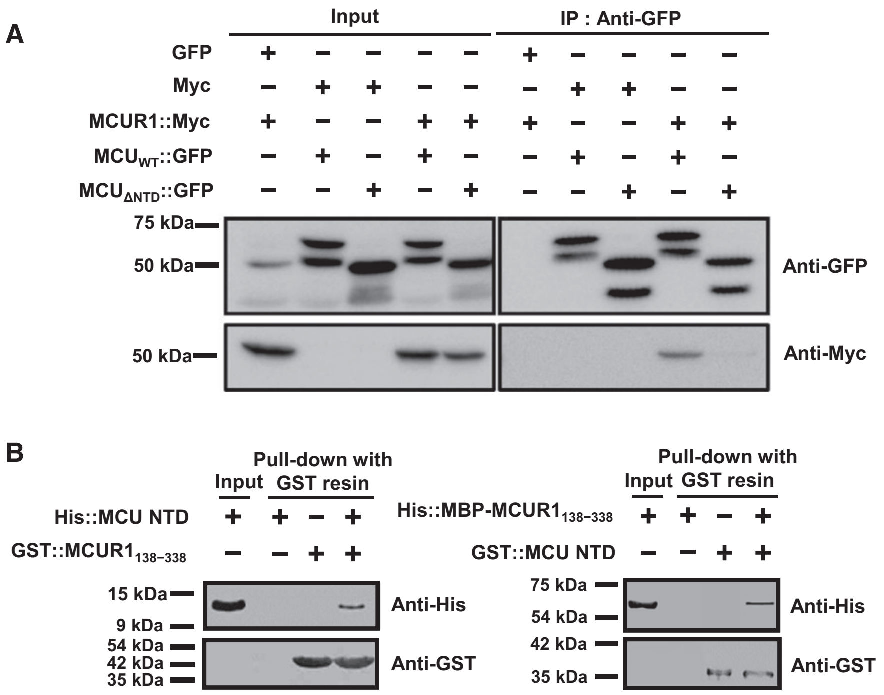

Figure EV5. Interaction of MCU NTD and MCUR1.

A HEK-293 FT cells were transiently co-transfected with myc-MCUR1 and MCU$_{WT}$-GFP or MCU$_{\Delta NTD}$-GFP. MCU$_{WT}$-GFP and MCU$_{\Delta NTD}$-GFP were precipitated from cell lysates with anti-GFP antibody. The precipitates were separated by SDS–PAGE and immunoblotted with the antibodies indicated. Co-immunoprecipitation of MCUR1 with MCU$_{\Delta NTD}$ was substantially diminished as compared to MCU$_{WT}$, suggesting that MCU NTD is necessary for interactions between MCU and MCUR1.

B *In vitro* pull-down assay of MCU NTD and MCUR1$_{138–338}$. His- or GST-tagged MCU NTD and MCUR1$_{138–338}$ were pulled down using GST affinity resin. The bait protients, GST-MCU NTD and GST-MCUR1$_{138–338}$, were immobilized on the GST resin, and then prey proteins, His-MCU NTD or His-MBP-MCUR1$_{138–338}$, flowed through the GST resin. The results were detected using Western blot after SDS–PAGE. The bait proteins, GST-MCUR1$_{138–338}$ and GST-MCU NTD, were pulled down with their respective prey proteins, His-MCU NTD and His-MBP-MCUR1$_{138–338}$, using GST affinity resin, as evidence for a direct interaction between MCU NTD and MCUR1$_{138–338}$. Thus, MCU NTD mediates the interaction between MCU and the side of MCUR1 facing the mitochondrial matrix.

© 2015 The Authors
